# Spontaneous Atraumatic Rupture of a Liver Hemangioma as a Rare Cause of Syncope

**DOI:** 10.1155/2024/7921410

**Published:** 2024-07-29

**Authors:** Fabian Sidler, Vitalie Turcan, Federico Storni, Sarah Bernhard, Dominik A. Jakob, Simone Ehrhard

**Affiliations:** ^1^ Department of General Internal Medicine Inselspital Bern University Hospital University of Bern, Bern, Switzerland; ^2^ Department of Visceral Surgery and Medicine Inselspital Bern University Hospital University of Bern, Bern, Switzerland; ^3^ University Clinic for Visceral Surgery and Medicine Inselspital Bern University Hospital, Bern, Switzerland; ^4^ Division of Angiology Swiss Cardiovascular Center Inselspital Bern University Hospital, Bern, Switzerland; ^5^ Vascular Center Bienna Hospital Center, Bienna, Switzerland; ^6^ Department of Emergency Medicine Inselspital Bern University Hospital, Bern, Switzerland

## Abstract

**Background:**

Syncope is common in emergency medicine, but only a minority of syncopes is caused by hemorrhage. Liver hemangioma is the most frequent benign liver tumor, and they rarely lead to symptoms or complications. *Case Presentation*. We describe the case of an 81-year-old man with hemorrhagic shock due to an atraumatic rupture of a hepatic hemangioma while on oral anticoagulation. The patient presented to the emergency department after three episodes of syncope before admission, nausea, vomiting, mild epigastric abdominal pain, but with clinical signs of peritonitis. On admission, the patient had a mild tachycardia with a heart rate of 107/min and a blood pressure of 102/83 mmHg. Initial hemoglobin was 122 g/L, and lactate was slightly elevated (2.5 mmol/L). Bedside sonography revealed free intraabdominal fluid. The subsequent computed tomography showed a ruptured hemangioma of the liver with ongoing hemorrhage. After the CT scan, the patient became increasingly tachycardic and the blood pressure dropped to 94/62 mmHg. After administration of blood products and intravenous fluids, the patient responded with improved hemodynamics and was transferred to angiology for emergency embolization. After the intervention, the patient spent two days in the intermediate care unit and was discharged after 10 days of hospitalization.

**Conclusion:**

Atraumatic rupture of a hemangioma with consecutive hemorrhagic shock is extremely rare. In selected cases of spontaneously ruptured hemangiomas with hemoperitoneum, endovascular embolization can be an alternative to surgery. Furthermore, this case emphasizes the importance of sonographic examination as an additional diagnostic tool in syncope and concomitant abdominal pain.

## 1. Introduction

Syncope is a common medical concern, with approximately 1.9% of all emergency department (ED) visits and up to 6% of all hospital admissions attributed to this condition, highlighting its significance in healthcare [1, 2]. More than 75% of persons older than 70 years will experience syncope at least once, with 20% suffering from two episodes or more [2]. Etiology of syncope is age-dependent [3] and has many underlying causes. Orthostatic hypotension due to hemorrhage is a rather rare cause [4]. An estimated 6.9% of all patients admitted with syncope experience, a nonfatal but severe outcome in the ED [5].

Hepatic hemangiomas are congenital vascular malformations and are considered the most frequent benign tumors of the liver with a prevalence of 0.4% to 20% in the general population [6]. While most hemangiomas are small (<4 cm) and solitary, they can reach up to 20 cm in diameter [6, 7]. Even large hemangiomas rarely cause symptoms [6]. Spontaneous rupture and intraperitoneal bleeding occurs in only 1–4% of hemangiomas and is an uncommon but severe complication with a reported mortality rate of 36–60% [8]. Surgical resection and enucleation have traditionally been considered the primary treatments for ruptured hemangiomas with persistent bleeding [9]. However, this approach has changed and endovascular embolization is increasingly used in the treatment of ruptured liver hemangiomas [10]. This case report presents a rare case of syncope due to hemorrhage under oral anticoagulation as a result of spontaneous rupture of a liver hemangioma.

## 2. Case Presentation

An 81-year-old patient was admitted by ambulance to the ED of the University Hospital Bern (Inselspital) with mild tachycardia, nausea accompanied by emesis, cold sweats, and three brief episodes of loss of consciousness without any associated trauma before admission. The patient's history revealed a two-day presence of epigastric pain. His past medical history included a syncope of unknown etiology three years ago and cholecystectomy with endoscopic retrograde cholangiopancreatography (ERCP) due to choledocholithiasis two years ago. In addition, the patient was known for arterial hypertension, atrial fibrillation, and dissection of both internal carotid arteries a few years ago, both treated conservatively. An unnoticed cardioembolic stroke was assumed due to parenchymal defect detected in cerebral imaging five years ago. In 2018, ultrasound and computed tomography of the abdomen demonstrated two liver hemangiomas. In 2020, the liver hemangioma in segment 4a/4b was size progressive from 40 mm to 56 mm in follow-up imaging. His daily medication consisted of rivaroxaban (20 mg/day) for atrial fibrillation, as well as candesartan and amlodipine for arterial hypertension.

The patient was evaluated on admission at the rescue bay of the ED. The respiratory rate was 24 breaths/minute with peripheral oxygen saturation of 98% while receiving two liters of oxygen/minute. He had a mild arrhythmic tachycardia (108 beats/min) with atrial fibrillation and a decreased blood pressure (102/83 mmHg) with a prolonged capillary refill time. The Glasgow coma scale was 15, the ear temperature was 35.2°C, and the documented body mass index was 24.5 kg/m^2^. Physical examination demonstrated diffuse abdominal tenderness and slight peritoneal irritation in the upper abdomen. The Extended Focused Assessment with Sonography in Trauma (E-FAST) was positive for intraperitoneal fluid in the splenorenal and hepatorenal recess as well as the rectovesical pouch. Laboratory values on admission are listed in Table 1.

**Figure 1:**
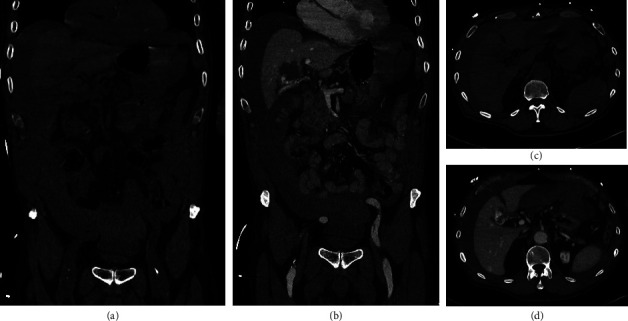
Computed tomography at admission. Computed tomography at admission is displayed. Frontal images of native (a) and venous (b) phase, as well as transverse images of native (c) and venous (d) phase, are shown.

To further evaluate the intraabdominal fluid, a computed tomography scan was conducted and revealed a ruptured hemangioma with a diameter of 40 mm in the liver segment 4a/4b with signs of persisting acute intraabdominal bleeding (venous pooling) (Figure 1). The laboratory showed a decrease in hemoglobin to 92 g/L.

**Figure 2:**
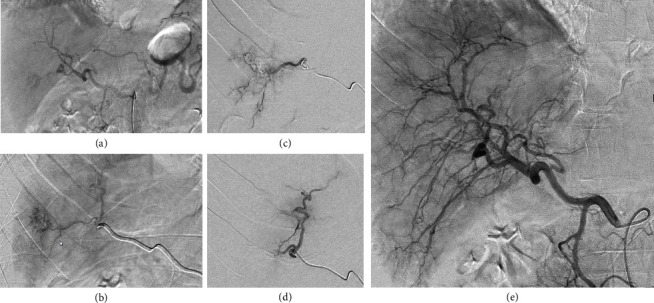
Digital subtraction angiography (DSA) during the intervention. DSA images of coeliacography (a) and selective angiography of the hemangioma (b) before treatment; selective angiography during particle embolization (c) and after embolization (d), as well as final coeliacography (e) phase, is shown.

With increasing hemodynamic instability in the time course (blood pressure 94/62 mmHg, 103 beats/minute), in addition to the total of 2000 ml of crystalloid fluids (given in boluses of 300−500 ml), two erythrocyte concentrates, two fresh frozen plasma, 1 g tranexamic acid, 1 g calcium gluconate, and low dose noradrenaline were administered. Anticoagulation was reversed with 2000 I.U. prothrombin complex concentrate. After stabilisation of the patient's vital signs (blood pressure 121/70 mmHg, 96 beats/minute) and in consultation with the colleagues from visceral surgery and angiology, the patient was transferred to angiology and a superselective particle embolization of the large hemangioma in segment 4a/4b was performed (Figure 2). After successful intervention, the patient was admitted to the intermediate care unit for two days and was then hospitalized on the visceral surgery ward for seven days before being discharged home. The anticoagulation therapy was temporarily switched to enoxaparine subcutaneous in a prophylactic dosage (40 mg 1*x*/d) three days after hemorrhage with increase to a subtherapeutic dosage (40 mg 2*x*/d) five days after hemorrhage for 7 days postdischarge followed by therapeutic anticoagulation with rivaroxaban. A magnetic resonance imaging 4.5 months after the bleeding event showed liver hemangiomas of constant size with posthemorrhagic changes of the ruptured liver hemangioma in segment 4a/4b.

## 3. Discussion

Liver hemangiomas are very common and rarely cause symptoms. According to the European Association for the Study of the Liver (EASL) Guidelines, there is no correlation between size of hemangiomas and complications [6]. Prophylactic surgical resection is therefore not broadly applicable, even in patients with extremely large hepatic hemangiomas [11].

Due to the benign course of hemangioma and the rare complications, the guidelines of the EASL and the American College of Gastroenterology (ACG) do not recommend follow-up imaging [6, 12]. However, patients with giant hemangiomas should be referred to a multidisciplinary team that specializes in benign liver tumors [6]. According to the ACG guidelines, surgical intervention in hemangiomas ≥10 cm should be taken into consideration [13], whereas the EASL guidelines only recommends intervention if hemangioma causes symptoms like pain or jaundice or if Kasabach–Merritt syndrome is present [6]. Since follow-up is not recommended, the focus is on the detection and the treatment of complications.

However, a retrospective Japanese study found a higher risk of rupture in patients with large (≥4 cm diameter) superficial or extrahepatic hemangiomas and concomitant steroid use [14]. Another retrospective study lists a size ≥4 cm, a peripheral or exophytic location, as a risk factor for rupture [15]. Despite the liver hemangioma size, none of the mentioned risk factors were present in our patient.

This patient received oral anticoagulation. This may have contributed to the onset of bleeding but could also have influenced the extent of bleeding that may have been more severe than in cases without anticoagulation. As only two cases of spontaneous rupture of a liver hemangioma under oral anticoagulation have been described in the literature, a meaningful statement of bleeding risk is very limited [16, 17]. However, the rivaroxaban level (result not available at the time of ED treatment) was below the trough level. Nevertheless, the administration of prothrombin complex concentrate may have had a positive effect on the coagulation status [18].

Although syncope is a common reason for ED admissions, some events are associated with an underlying condition that is associated with increased mortality. Patient's history and a structured clinical examination are crucial for identifying severe underlying conditions and guarantee a resource-saving approach. Bedside sonography can be used as an additional tool to quickly rule out abdominal hemorrhage in patients presenting after loss of consciousness with abdominal pain.

## Figures and Tables

**Table 1:** Laboratory parameter on emergency department admission, 75 minutes after admission and after CT scan^3^.

Parameter	Normal values	On admission (*T* = 0)	After admission (*T* = +75 minutes)	After CT scan (*T* = +113 minutes)
Hemoglobin (g/L)	135–168	122	92	104
Erythrocytes (10^3^/L)	4.2–5.7	3.84	n.a.	3.36
Thrombocytes (G/L)	150–450	302	n.a.	211
INR^1^	0.7–1.2	1.30	n.a.	1.19
aPTT^2^ (second)	25–36	33.1	n.a.	36.7
Lactate (mmol/L)	0.63–2.44	2.5	1.5	1.5
Rivaroxaban (ng/ml)		n.a.	n.a.	124.81

^1^International normalised ratio (INR). ^2^Activated partial thromboplastin time (aPTT). ^3^Computed tomography.

## Data Availability

The data used to support the findings of the study are available from the corresponding author upon request.
